# Searching for Factors Influencing the Severity of the Symptoms of Long COVID

**DOI:** 10.3390/ijerph19138013

**Published:** 2022-06-30

**Authors:** Alicja Mińko, Agnieszka Turoń-Skrzypińska, Aleksandra Rył, Natalia Tomska, Zuzanna Bereda, Iwona Rotter

**Affiliations:** 1 Department of Medical Rehabilitation and Clinical Rehabilitation, Pomeranian Medical University, 71-210 Szczecin, Poland; agi.skrzypinska@gmail.com (A.T.-S.); ryl.ola@gmail.com (A.R.); natalia.tomska@o2.pl (N.T.); iwrot@wp.pl (I.R.); 2 Student Science Club “KINEZIS”, Department of Medical Rehabilitation and Clinical Physiotherapy, Pomeranian Medical University, 71-210 Szczecin, Poland; zuzanna.bereda@gmail.com

**Keywords:** COVID-19, SARS-CoV-2, Long COVID, BMI

## Abstract

COVID-19 is a highly contagious respiratory disease. Infection with the virus can occur with differing symptom severity, from mild and moderate to severe cases, but the long-term consequences of infection have not been fully identified or studied. Long COVID is defined as occurring in individuals with a history of probable or confirmed SARS-CoV-2 infection, and symptoms persisting for at least two months within three months of onset that cannot be explained by an alternative diagnosis. The purpose of this study was to look for factors that influence the type and severity of Long COVID symptoms. In total, 932 individuals with a history of COVID-19 were qualified for the study using an original questionnaire based on the COVID-19 Yorkshire Rehab Screen (C19-YRS) questionnaire. Older adults were more likely to report problems with mobility (*p* < 0.001) and in performing daily activities (*p* = 0.014). Those with a higher BMI showed significantly more symptoms such as dyspnea at rest (*p* < 0.001) and on exertion (*p* < 0.001), feelings of chronic fatigue (*p* = 0.023), problems with mobility (*p* < 0.001), and in performing daily activities (*p* = 0.002). The data show that those with Long COVID should receive multidisciplinary help including additional medical and psychological support. Particular attention should be paid to elderly and obese persons, who should be included in rehabilitation programs after COVID-19 in the first place.

## 1. Introduction

COVID-19 is a highly contagious respiratory disease caused by a virus belonging to the CoV β species—SARS-CoV-2. It is the third deadliest coronavirus, after SARS-CoV-1 (2003), which causes severe acute respiratory syndrome (SARS), and MERS-CoV (2012), which causes Middle East respiratory syndrome (MERS) [1,2]. The first case of SARS-CoV-2 was observed in 2019 in the city of Wuhan in the Chinese province of Habei. Its rapid spread led to the declaration of a global pandemic by the World Health Organization (WHO) on 1 March 2020 [3,4,5]. Infection with the virus can occur with differing symptom severity, from mild and moderate to severe cases, requiring hospitalization and the use of mechanical ventilation [6,7]. The prognosis for patients is not fully understood, and the type and severity of the consequences of COVID-19 may depend, inter alia, on the severity of the course of the infection, age, and comorbidities. Various reports show that health problems following COVID-19 can persist for up to a year after recovery [8,9,10,11].

Long COVID is defined as a disease occurring in individuals with a history of probable or confirmed SARS-CoV-2 infection and symptoms persisting for at least two months within three months of onset, where the symptoms and effects of Long COVID cannot be explained by an alternative diagnosis. In the available literature, the term Long COVID is often replaced by the term Post-COVID. Although there is much debate on the subject, according to recent literature these two terms should not be confused. The term Post-COVID includes symptoms that do not appear until 12 weeks after SARS-CoV-2 infection [12,13]. The most common symptoms are fatigue and dyspnea, cognitive disorders, and pain in muscles and joints. Potential factors that influence Long COVID may be related to pathological inflammation, organ damage, non-specific effects of hospitalization or prolonged ventilation (post-intensive care syndrome), social isolation, or the impact on pre-existing medical conditions. Long COVID can affect COVID-19 survivors at any severity of the disease. Studies have shown that Long COVID syndrome affects even mild cases of SARS-CoV-2 infection. Previous studies suggest that the risk of Long COVID is twice as common in women compared to men. Increasing age is also a risk factor. The presence of more than five symptoms in the acute stage of the disease is also associated with an increased risk of developing Long COVID. In order to define appropriate prevention and intervention strategies following COVID-19, it seems important to understand the possible long-term consequences of COVID-19. More research is needed to identify potential risk factors for developing Long COVID syndrome [12,14,15,16,17,18,19,20,21,22].

The purpose of this study was to look for factors that influence the type and severity of Long COVID symptoms.

## 2. Materials and Methods

### 2.1. Survey Sample

**Figure 1:**
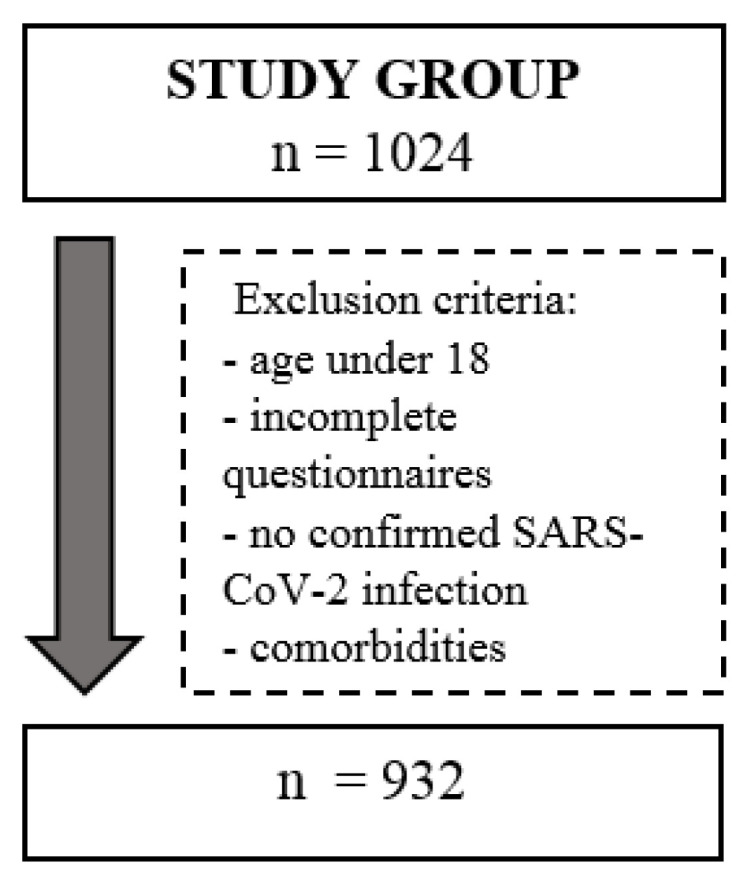
Exclusion criteria.

In total, 1024 Polish individuals with a history of SARS-CoV-2 infection took part in a prospective study conducted from May 2020 to December 2021. The inclusion criteria for the study included a history of SARS-CoV-2 infection, as confirmed by a positive PCR test, an age of at least 18, and a fully completed questionnaire (Figure 1). Minors and those without confirmed SARS-CoV-2 infection were excluded from the study, as were those who only completed part of the questionnaire. Known specific health problems also disqualified individuals from participation in the study, such as the coexistence of rheumatoid diseases, cancer, post-stroke or myocardial infarction, and any other health problems occurring in the period before COVID-19 infection that could affect functional assessment. Ultimately, considering all the inclusion and exclusion criteria, 932 individuals were qualified for the study. The participants were recruited through online forums associating persons with a history of COVID-19 infection. As a result of the study’s online recruitment, study participants self-reported the presence of long-term COVID-19 symptoms.

### 2.2. Questionnaires

The study was conducted using an original questionnaire that was based on the COVID-19 Yorkshire Rehab Screen (C19-YRS) questionnaire, and included sociodemographic questions (gender, age, height, weight, place of residence, comorbidities, the occurrence of addictive behaviors), questions about the course of infection (date of infection, need for hospitalization, the occurrence of pneumonia, need for mechanical ventilation), and current complications.

In the section on current complications, questions were asked about shortness of breath at rest and during exercise, respiratory tract problems (coughing, loud breathing), mobility problems (moving and walking), eating problems (loss of appetite, dysosmia, and dysgeusia), chronic fatigue, muscle and joint pain, and problems with everyday activities (washing, getting dressed, working at home). Additionally, questions were asked about the occurrence of symptoms disturbing mental comfort (unwanted memories, thoughts, feelings related to the disease). The respondents were also asked to define their perception of their health.

The respondents answered each question on a scale from 0 to 10. The respondents answered in relation to their current COVID-19 state and the state before the SARS-CoV-2 infection.

Nutritional status was determined on the basis of Body Mass Index (BMI). According to WHO guidelines, a score of 17–18.49 indicates underweight. The range 18.5–24.99 is considered the norm. The ranges 25.0–29.99, 30.0–34.99, 35.0–39.99, and scores ≥ 40.0 indicates overweight, 1st degree obesity, 2nd degree obesity, and 3rd degree obesity, respectively.

The study was conducted in accordance with the standards of the Helsinki Declaration. It has been approved by the Bioethics Committee of the Pomeranian Medical University (decision no KB-0012/87/06/2021/Z).

### 2.3. Statistical Analysis

Statistical analysis was performed with StatSoft Inc. STATISTICA v 13.1 and MS Excel spreadsheets. The minimum sample size was 385. The study group was characterized in terms of the number of patients, their percentage share, mean, minimum and maximum, and standard deviation. The normality of distribution was tested with a Shapiro-Wilk test. The relationships between the groups were analyzed with the Kruskal-Wallis test. The relationships between the groups were performed with the post hoc analysis. Correlation analysis was performed with the Spearman’s ρ test.

## 3. Results

### 3.1. Group Characteristics

A total of 932 individuals were included in the study. The subjects were characterized in terms of age, gender, nutritional status, and place of residence. The analyzed factor was the need for hospitalization during the course of COVID-19. The mean age in the study group was 40.44 (±11.53). The mean height and weight of the study population were 167.84 (±7.96) and 81.9 (±231.05), respectively. The mean body mass index (BMI) was 25.51 (±4.72). The characteristics of the study population are presented in Table 1.

### 3.2. Correlations between Complications after COVID-19 and Individual Variables

The analysis of the results is presented in the form of correlations between individual complications following COVID-19 and factors such as time from the infection, age, and nutritional status.

The severity of persistent symptoms following COVID-19 decreased significantly with the time after the infection for shortness of breath during exercise (*p* < 0.001), problems with mobility (*p* = 0.010) and with everyday activities (*p* = 0.012), as well as pain in muscles and joints (*p* = 0.029).

Older individuals significantly more often reported problems with mobility (*p* < 0.001) and problems with performing everyday activities (*p* = 0.014). Lower age was associated with intensification of mental health disorders (*p* = 0.005).

Among those with higher BMI, significant intensification of symptoms was observed, such as shortness of breath at rest (*p* < 0.001) and during exercise (*p* < 0.001), feelings of chronic fatigue (*p* = 0.023), and problems with mobility (*p* < 0.001) and with everyday activities (*p* = 0.002). Those with higher BMI perceived their health to be worse (*p* = 0.002). Detailed results are presented in Table 2.

Table 3 shows the relationship between complications following COVID-19 and individual BMI levels. The statistically significant variables included the occurrence of shortness of breath at rest (*p* = 0.042) and during exercise (*p* < 0.001), as well as problems with mobility (*p* < 0.001).

The relationships between complications following COVID-19 and time from the infection are presented in Table 4. With more time following SARS-CoV-2 infection, the severity of Long COVID symptoms decreased. The statistically significant variables included occurrence of shortness of breath during exercise (*p* = 0.003), problems with mobility (*p* = 0.015), and problems in performing everyday activities (*p* < 0.001).

Table 5 shows the relationship between complications following COVID-19 and gender. The statistically significant variables included occurrence of shortness of breath during exercise (*p* = 0.029), coughing (*p* = 0.002), problems with mobility (*p* = 0.015), pain in muscles and joints (*p* = 0.012), and disturbances in mental health comfort (*p* < 0.001).

A significant correlation was demonstrated between the severity of symptoms before and after COVID-19 infection. The individuals described symptoms such as the occurrence of shortness of breath at rest and during exercise, the occurrence of chronic fatigue and pain in muscles and joints, as well as problems with moving and performing everyday activities. Correlations were also demonstrated for disturbance in mental comfort. Detailed results are presented in Table 6.

## 4. Discussion

There are many studies in available literature describing possible complications after the infection with SARS-CoV-2. However, the factors contributing to the severe or long-lasting symptoms of the infection are still unknown. This study is a multivariate analysis that assessed the relationship between complications after COVID-19 and variables such as time from the infection, age, gender, and BMI.

The impact of COVID-19 on the human body is still not fully understood. It is also not known what sustained strain on the body the infection may cause. The latest research led to the description of a new term, Long COVID syndrome. It has been defined as the occurrence of various physical and mental symptoms for more than 12 weeks after the infection with SARS-CoV-2, with no alternative explanation other than a history of COVID-19 infection [23,24,25,26,27]. More and more authors in their research focus on understanding the pathophysiology and risk factors of the new term [28,29,30].

The pathophysiology of Long COVID is not fully understood. Some studies suggest that symptoms of Long COVID are caused by endothelial dysfunction and may be complicated by microthrombosis. Another theory argues that the course of SARS-CoV-2 infection causes immune dysregulation, resulting in the development of Long COVID. Other studies report that Long COVID is associated with a disruption of the autonomic nervous system mediated by the virus or the immune system, resulting in orthostatic intolerance syndromes [31,32,33].

Dyspnea and muscle pain are the most frequently reported symptoms after COVID-19. Other common symptoms are extreme fatigue, depressed mood, and sleep disturbances [34,35,36,37,38]. Dicpinigaitis et al. show that respiratory symptoms such as coughing or noisy breathing are not common symptoms in patients with Long COVID [39], as confirmed by other authors [40,41].

Sykes et al. compared Long COVID to Chronic Fatigue Syndrome (CFS), which is diagnosed in the presence of symptoms such as post-exertional fatigue, cognitive difficulties, sleep disturbances, and chronic pain. However, symptoms would need to persist beyond 4 months. They noticed clear epidemiological similarities and emphasized the need to integrate services provided for patients with CFS (symptom management, psychological treatments, employment support, and health education) into health services for patients with Long COVID [42].

The exact risk factors for the development of Long COVID syndrome are not known. There is evidence that there is little correlation between the severity of acute disease and the likelihood of developing Long COVID [43,44]. Our research shows that women, the elderly, and obese people are more exposed to the development of chronic COVID-19. Researchers in other studies obtained the same results [45,46,47,48,49,50,51,52]. Women reported depressed mood, muscle pain, fatigue, and sleep disturbances significantly more often than men [46,52].

Other reports have shown that there is a relationship between age and the development of Long COVID. Older persons were more prone to persistent symptoms after infection [20,53,54]; however, Akbarialiabadi et al. reported that persistent anosmia and taste disturbances were associated with a lower age (<65 years) [17].

Obesity is also a factor associated with greater symptoms after COVID 19 [49,50,51]. In this study, a relationship was found between BMI and symptoms such as dyspnea at rest and during exercise, as well as problems in performing everyday activities. In a study by Sykes et al., higher BMI was associated with muscle pain and fatigue [42]. The relationship between higher BMI and greater post-viral symptoms has also been demonstrated by other researchers [55,56]. It is worth noting that obesity is a strong risk factor for the development of a number of comorbidities that are associated with increased morbidity and mortality. Therefore, those with a higher BMI are at risk of a more severe recovery process, not only after COVID-19 infection. This is related to, inter alia, a defective response in both innate and acquired immunity. For this reason, special attention should be paid to patients with high BMI [57,58,59].

Long-term symptoms of COVID-19 are also associated with mental health. According to our research, age was associated with greater susceptibility to disturbances in mental comfort. Women were also more prone to developing mental disorders. Similar observations are reported by other researchers who have proved in their research that female gender and older age were the most common risk factors for mental health [42,45,51]. According to this study, other reports have shown that long-term mental health problems were also associated with a more severe course of COVID-19 [60,61,62].

Further research is still required to define the factors that influence subsequent complications after COVID-19. Our study highlights the long-term impact COVID-19 could have on patients.

### Limitations

Due to the ongoing SARS-CoV-2 coronavirus pandemic, direct access to study subjects was difficult, and they were ultimately approached via online forums for SARS-CoV-2 survivors. Study subjects were self-reported COVID-19 survivors based on PCR-confirmed SARS-CoV-2 infection and the presence of chronic symptoms following COVID-19. It was not possible to exclude an alternative diagnosis of Long COVID; therefore, the study population may not completely represent patients with Long COVID. As a result of the respondents filling in the online questionnaires themselves, there was a risk of an information error. It was not possible to check and, if necessary, correct the answers to individual questions. In addition, respondents wrote about the experiences they had several weeks or months earlier; thus, the information could be distorted by memory errors. A limitation of our study was also the inability to record any physiological data.

## 5. Conclusions

Factors that may be associated with the severity of Long COVID symptoms include age, nutritional status, time since infection expressed by body mass index (BMI), and sex. People with Long COVID should receive multidisciplinary help, which would include additional medical and psychological support. Particular attention should be paid to the elderly and obese people, who should be implemented in rehabilitation programs after COVID-19 in the first place.

## Figures and Tables

**Table 1:** Characteristics of the study group.

Variable		n	%
**Gender**	female	786	84.33
	male	146	15.67
**Age**	18–25 years	86	9.23
	26–35 years	244	26.18
	36–45 years	325	34.87
	46–55 years	169	18.13
	56–65 years	83	8.91
	over 60 years	25	2.68
**Place of residence**	village	196	21.03
	city up to 10 thousand	37	3.97
	city from 10 to 50 thousand	159	17.06
	city with 50 to 100 thousand	91	9.76
	city with over 100 thousand	449	48.18
**Hospitalization**	yes	85	9.12
	no	847	90.88
**Nutritional status (BMI)**	17.0–18.49 (underweight)	108	11.59
	18.5–24.99 (norm)	351	37.66
	25.0–29.9 (overweight)	269	28.86
	30.0–34.99 (1st degree obesity)	136	14.59
	35.0–39.99 (2nd degree obesity)	50	5.36
**Time from SARS-CoV-2 infection**	up to 3 months	399	42.81
	from 3 to 6 months	125	13.41
	over 6 months	408	43.78

Legend: n—number, BMI—body mass index.

**Table 2:** Correlation between the time since infection, age and nutritional status, and complications after COVID-19.

Pair of Variables		R	*p*
**Time since SARS-CoV-2 infection**	Shortness of breath at rest	−0.050792	0.121
	Shortness of breath during exercise	−0.112570	<0.001 *
	Coughing/loud breathing	−0.028147	0.505
	Problems with mobility	−0.083910	0.010 *
	Fatigue	−0.007824	0.831
	Muscle/joint pain	−0.071215	0.029 *
	Eating problems	−0.075123	0.101
	Problems with everyday activities	−0.081740	0.012 *
	Disturbances in mental comfort	−0.023006	0.482
	Overall health perception	0.064012	0.050 *
**Age**	Shortness of breath at rest	0.026036	0.427
	Shortness of breath during exercise	−0.001883	0.954
	Coughing/loud breathing	0.041950	0.320
	Problems with mobility	0.172705	<0.001 *
	Fatigue	0.029857	0.416
	Muscle/joint pain	−0.013400	0.683
	Eating problems	−0.082353	0.073
	Problems with everyday activities	0.080511	0.014 *
	Disturbances in mental comfort	−0.091730	0.005 *
	Overall health perception	−0.004706	0.886
**BMI**	Shortness of breath at rest	0.111565	<0.001 *
	Shortness of breath during exercise	0.150855	<0.001 *
	Coughing/loud breathing	0.023634	0.584
	Problems with mobility	0.134040	<0.001 *
	Fatigue	0.085030	0.023 *
	Muscle/joint pain	0.023721	0.478
	Eating problems	−0.066431	0.158
	Problems with everyday activities	0.103615	0.002 *
	Disturbances in mental comfort	−0.001368	0.967
	Overall health perception	−0.104278	0.002 *

Legend: *p*—statistical significance *; R—correlation coefficient.

**Table 3:** Relationship between BMI levels and complications after COVID-19.

Variable	Underweight			Normal Weight			Overweight			1st Degree Obesity			2nd Degree Obesity			*p*
	M	Min–Max	SD	M	Min–Max	SD	M	Min–Max	SD	M	Min–Max	SD	M	Min–Max	SD	
**Shortness of breath at rest**	1.48	0.0–10.0	2.49	1.42	0.0–10.0	2.16	1.68	0.0–10.0	2.50	2.13	0.0–10.0	2.64	2.00	0.0–9.0	2.62	0.042 *
**Shortness of breath during exercise**	3.04	0.0–10.0	2.86	3.29	0.0–10.0	2.91	3.59	0.0–10.0	2.97	4.23	0.0–10.0	3.11	4.19	0.0–9.0	2.97	<0.001 *
**Coughing/loud breathing**	4.49	0.0–10.0	3.08	4.05	0.0–10.0	3.06	4.20	0.0–10.0	3.07	4.21	0.0–10.0	3.26	4.90	1.0–10.0	2.69	0.574
**Problems with mobility**	1.41	0.0–9.0	2.20	1.36	0.0–10.0	2.27	1.70	0.0–10.0	2.32	2.07	0.0–10.0	2.60	1.64	0.0–8.0	2.13	<0.001 *
**Fatigue**	3.86	0.0–10.0	3.05	3.74	0.0–10.0	2.88	4.01	0.0–10.0	2.82	4.40	0.0–10.0	3.24	4.41	0.0–10.0	2.49	0.321
**Muscle/joint pain**	2.27	0.0–10.0	2.98	1.98	0.0–9.0	2.35	2.11	0.0–10.0	2.59	2.33	0.0–10.0	2.74	1.89	0.0–8.0	2.41	0.930
**Eating problems**	4.13	0.0–10.0	3.28	4.23	0.0–10.0	3.23	3.89	0.0–10.0	3.06	4.14	0.0–10.0	3.46	2.62	0.0–9.0	3.01	0.371
**Problems with everyday** **activities**	2.29	0.0–10.0	2.87	2.31	0.0–10.0	2.49	2.41	0.0–10.0	2.65	2.73	0.0–10.0	2.73	2.19	0.0–8.0	2.45	0.454
**Disturbances in mental comfort**	3.83	0.0–10.0	3.40	3.21	0.0–10.0	3.02	2.96	0.0–10.0	3.03	3.80	0.0–10.0	3.36	3.42	0.0–10.0	3.33	0.099
**Overall health perception**	5.32	0.0–10.0	3.09	4.74	0.0–10.0	2.65	4.61	0.0–10.0	2.69	4.38	0.0–10.0	2.67	4.22	0.0–10.0	2.45	0.137

Legend: *p*—statistical significance *, M—arithmetic mean; SD—standard deviation; Min—minimum; Max—maximum; BMI—body mass index.

**Table 4:** Relationship between the time since the infection and complications after COVID-19.

Variable	<3 Months			3–6 Months			>6 Months			*p*
	M	Min–Max	SD	M	Min-Max	SD	M	Min–Max	SD	
**Shortness of breath at rest**	1.76	0.0–10.0	2.47	1.90	0.0–10.0	2.66	1.51	0.0–10.0	2.34	0.245
**Shortness of breath during exercise**	1.71	0.0–10.0	2.18	1.76	0.0–9.0	2.40	1.34	0.0–10.0	2.12	0.003 *
**Coughing/loud breathing**	4.29	0.0–10.0	3.04	4.49	0.0–10.0	3.28	4.08	0.0–10.0	3.08	0.579
**Problems with mobility**	1.70	0.0–10.0	2.31	2.10	0.0–10.0	2.82	1.39	0.0–10.0	2.22	0.015 *
**Fatigue**	4.01	0.0–10.0	2.98	4.10	0.0–10.0	3.05	3.93	0.0–10.0	2.87	0.920
**Muscle/joint pain**	2.22	0.0–10.0	2.59	2.40	0.0–9.0	2.94	1.88	0.0–10.0	2.41	0.078
**Eating problems**	2.40	0.0–10.0	3.18	3.15	0.0–10.0	3.29	2.19	0.0–10.0	3.25	0.221
**Problems with everyday activities**	2.58	0.0–10.0	2.67	0.95	0.0–10.0	2.04	0.64	0.0–10.0	1.61	<0.001 *
**Disturbances in mental comfort**	2.32	0.0–10.0	2.77	2.93	0.0–10.0	3.02	2.48	0.0–10.0	2.98	0.175
**Overall health perception**	4.48	0.0–10.0	2.69	4.90	0.0–10.0	2.64	4.86	0.0–10.0	2.77	0.099

Legend: *p*—statistical significance *, M—arithmetic mean; SD—standard deviation; Min—minimum; Max—maximum.

**Table 5:** Relationship between gender and complications after COVID-19.

Variable	Female			Male			*p*
	M	Min–Max	SD	M	Min–Max	SD	
**Shortness of breath at rest**	1.69	0.0–10.0	2.45	1.50	0.0–9.0	2.34	0.248
**Shortness of breath during exercise**	3.60	0.0–10.0	2.98	3.13	0.0–10.0	2.98	0.029 *
**Coughing/loud breathing**	4.39	0.0–10.0	3.06	3.31	0.0–10.0	3.06	0.002 *
**Problems with mobility**	1.62	0.0–10.0	2.35	1.57	0.0–10.0	2.31	0.015 *
**Fatigue**	4.00	0.0–10.0	2.91	3.85	0.0–10.0	3.08	0.507
**Muscle/joint pain**	2.16	0.0–10.0	2.57	1.70	0.0–9.0	2.49	0.012 *
**Eating problems**	4.12	0.0–10.0	3.18	3.38	0.0–10.0	3.48	0.081
**Problems with everyday activities**	0.89	0.0–10.0	1.89	0.76	0.0–7.0	1.64	0.571
**Disturbances in mental comfort**	3.48	0.0–10.0	3.18	2.47	0.0–10.0	2.96	<0.001 *
**Overall health perception**	4.73	0.0–10.0	2.66	4.55	0.0–10.0	2.99	0.383

Legend: *p*—statistical significance *, M—arithmetic mean; SD—standard deviation; Min—minimum; Max—maximum.

**Table 6:** Correlation of the severity of symptoms before and after COVID-19.

Pair of Variables		*p*
**Difference between** **before and after** **COVID-19**	Shortness of breath at rest	<0.001 *
	Shortness of breath during exercise	<0.001 *
	Coughing/loud breathing	<0.001 *
	Problems with mobility	<0.001 *
	Fatigue	<0.001 *
	Problems with everyday activities	<0.001 *
	Disturbances in mental comfort	<0.001 *
	Overall health perception	0.534

Legend: *p*—statistical significance *.

## Data Availability

All data were collected in the Department of Medical Rehabilitation and Clinical Rehabilitation, Pomeranian Medical University, 71-210 Szczecin.
